# Utilization of Human Samples for Assessment of Mitochondrial Bioenergetics: Gold Standards, Limitations, and Future Perspectives

**DOI:** 10.3390/life11090949

**Published:** 2021-09-10

**Authors:** Rebeca Acin-Perez, Cristiane Benincá, Byourak Shabane, Orian S. Shirihai, Linsey Stiles

**Affiliations:** 1 Department of Medicine, Endocrinology, David Geffen School of Medicine, University of California, Los Angeles, CA 90095, USA; CBeninca@mednet.ucla.edu (C.B.); BShabane@mednet.ucla.edu (B.S.); OShirihai@mednet.ucla.edu (O.S.S.); 2 Metabolism Theme, David Geffen School of Medicine, University of California, Los Angeles, CA 90095, USA; 3 Department of Molecular and Medical Pharmacology, University of California, Los Angeles, CA 90095, USA; 4 Molecular Biology Institute, University of California, Los Angeles, CA 90095, USA

**Keywords:** bioenergetics, fibroblasts, frozen tissue, leukocytes, mitochondria, oxygen consumption, platelets, respirometry, skeletal muscle

## Abstract

Mitochondrial bioenergetic function is a central component of cellular metabolism in health and disease. Mitochondrial oxidative phosphorylation is critical for maintaining energetic homeostasis, and impairment of mitochondrial function underlies the development and progression of metabolic diseases and aging. However, measurement of mitochondrial bioenergetic function can be challenging in human samples due to limitations in the size of the collected sample. Furthermore, the collection of samples from human cohorts is often spread over multiple days and locations, which makes immediate sample processing and bioenergetics analysis challenging. Therefore, sample selection and choice of tests should be carefully considered. Basic research, clinical trials, and mitochondrial disease diagnosis rely primarily on skeletal muscle samples. However, obtaining skeletal muscle biopsies requires an appropriate clinical setting and specialized personnel, making skeletal muscle a less suitable tissue for certain research studies. Circulating white blood cells and platelets offer a promising primary tissue alternative to biopsies for the study of mitochondrial bioenergetics. Recent advances in frozen respirometry protocols combined with the utilization of minimally invasive and non-invasive samples may provide promise for future mitochondrial research studies in humans. Here we review the human samples commonly used for the measurement of mitochondrial bioenergetics with a focus on the advantages and limitations of each sample.

## 1. Introduction

Mitochondrial function is essential to meet energy demand and coordinate cellular function. Mitochondria are often referred to as the powerhouse of the cell. However, in addition to converting nutrients into energy that can be used by the cell, mitochondria also play an important role in intercellular signaling, calcium buffering, biosynthesis, and apoptosis [1]. Consumed nutrients get broken down into small molecules that can be used either to enter catabolic or anabolic processes. In the catabolic processes, nutrients break down into small molecules that are used to fuel the mitochondrial electron transport chain (ETC) to either produce adenosine triphosphate (ATP) through oxidative phosphorylation (OXPHOS) or generate heat. On the other hand, the anabolic processes involve building up molecules that can be used by the cells as storage or for structural purposes. The balance between catabolism and anabolism defines the metabolic signature in every tissue [2,3,4,5]. Therefore, mitochondrial metabolism is critical for maintaining energetic homeostasis and mitochondrial dysfunction promotes the development and progression of metabolism- and aging-related disorders.

**Figure 1:**
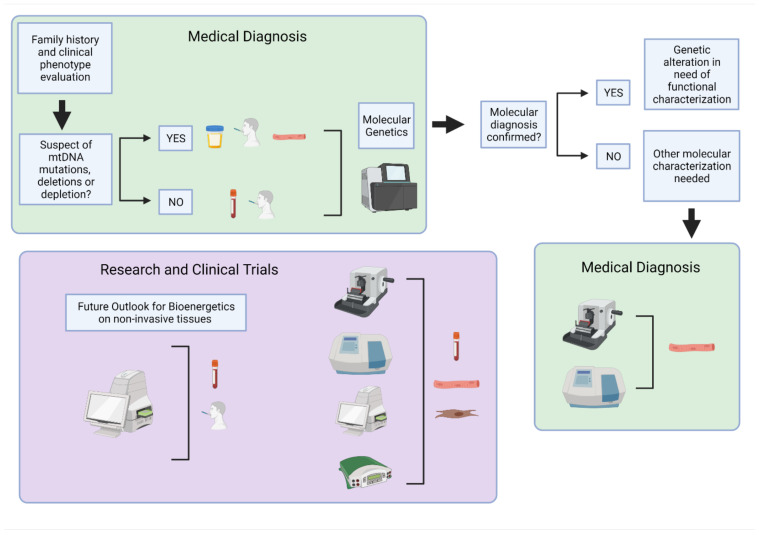
Overview of human samples and bioenergetic assessment used for clinical diagnosis and research. The primary approach for medical diagnosis of mitochondrial disorders (green boxes) is genetic testing of non-invasive or minimally invasive samples. Follow-up testing may be required in skeletal muscle biopsies when additional molecular characterizations are needed for diagnosis or to determine the extent of mitochondrial dysfunction. These include histology and biochemical analysis. For mitochondrial function evaluation in research (purple box), skeletal muscle, primary fibroblasts, and circulating blood samples are routinely used for molecular and biochemical analysis, histology, and bioenergetic testing. Further optimization and development of non-invasive samples may provide a future path for expanding bioenergetics research in human subjects to include large-scale population studies and clinical trials. Created with BioRender.com on 10 August 2021.

Mitochondrial diseases are genetically inherited, clinically heterogeneous disorders mainly affecting post-mitotic tissues with high energy demand, such as the brain, heart, and skeletal muscle. Since symptoms are varied, affecting multiple organs in the body, diagnosing mitochondrial disease can be challenging. Genetic testing (Figure 1) is the most reliable way to diagnose and categorize a mitochondrial disorder but requires the right choice of starting material and sequencing technique [6]. Specifically, for mitochondrial deoxyribonucleic acid (mtDNA) encoded diseases, the variability observed by mutational heteroplasmy and copy number in different tissues needs to be considered. A negative genetic result does not exclude a mtDNA-related disorder and sampling of tissues such as the urinary tract and buccal epithelial cells or skeletal muscle is often needed. Moreover, some mtDNA variants, specifically deletions and depletion-causing mutations, are detectable only in the high-energy demand affected tissue [7]. Therefore, even with genetic testing available, molecular diagnosis often requires functional characterization. For that, muscle biopsy is the gold standard sample to perform enzymatic activities and/or histology in clinical settings. In research, besides the use of muscle biopsies, skin biopsy-derived fibroblasts are also used for functional characterization of mitochondrial disorders with newly discovered mutations and genes. In this case, assessing the ETC function is required for analysis of mitochondrial-dependent cell metabolism with measuring oxygen consumption, namely respirometry, as the gold standard measurement of mitochondrial function. Respirometry provides an integrated measurement of oxidative mitochondrial metabolism. Platforms and applications for measuring respiration in fresh samples are well established and have been extensively described and reviewed [8,9,10,11,12,13,14,15,16,17,18,19,20,21,22,23,24].

However, measuring respiration in primary tissue samples from humans can be challenging given that a muscle biopsy may not be accessible, sample amount may be limiting, and, when measuring respiration using the traditionally established protocols, there is the limitation that fresh tissue and extensive processing is required. This is due to the fact that freeze-thawing samples damages membranes resulting in cytochrome c release from the mitochondrial intermembrane space, which makes the electron transport chain inefficient as cytochrome c is an essential electron carrier. In addition, damage to mitochondrial membranes causes respiration to uncouple energy production. For these reasons, respirometry must be performed quickly and is done primarily on-site, which limits the use of respirometry as a diagnostic approach, for use in clinical trials, and in previously frozen samples stored in biobanks. These limitations make translational mitochondrial research challenging and largely unfeasible on a large scale. To address these challenges, new approaches are emerging to make use of existing samples for respirometry and use less invasive samples, which will be discussed later in this review.

## 2. Materials and Methods

### 2.1. Cell Culture

Lymphoblastoid cell lines were cultured according to the manufacturer’s instructions in Roswell Park Memorial Institute (RPMI)-1640 medium supplemented with 2 mM glutamine and 15% fetal bovine serum (FBS). Frozen LCLs went through three freeze-thaw cycles with liquid nitrogen before use in enzymatic assays or respirometry. Lymphocytes were cryopreserved and did not require cell culture. Thawed lymphocytes were treated with 0, 0.1, 0.5, or 1 µM rotenone for 10 min and then frozen at −80 °C.

### 2.2. Material and Reagents

Table 1 presents all the reagents with the associated supplier and catalog number that were used to generate data for this review.

### 2.3. XF96 Extracellular Flux Analysis in Fresh and Frozen Cells

In all respirometry assays, lymphocytes and LCLs were spun down on poly-d-lysine-coated plates on the day of the assay. LCLs were plated at 200,000 cells per well and lymphocytes were plated at 400,000 cells/well. Cells were added to the plate at a total volume of 175 µL per well and centrifuged at 400× *g* for 5 min with no break. Table 2 presents the mitochondrial-targeted compounds and substrates used and the associated mechanism of action.

#### 2.3.1. Intact Cell Respirometry

Intact lymphocytes respirometry was measured in a DMEM assay medium containing 5 mM glucose, 2 mM glutamine, 1 mM pyruvate, and 5 mM HEPES. Injections resulted in a final concentration of 2 µM oligomycin from Port A, 3 µM FCCP from Ports B and C, and 2 µM antimycin A and rotenone from Port D. Measurements included three basal measurements, three measurements after injection of oligomycin, four FCCP measurements, and three antimycin A and rotenone measurements. Respiration measurements were conducted with four technical replicates.

#### 2.3.2. Permeabilized Cell Respirometry

Permeabilized lymphocytes respirometry was measured in Mitochondrial Assay Solution (MAS) (70 mM Sucrose, 220 mM Mannitol, 5 mM KH_2_PO_4_, 5mM MgCl_2_, 1 mM EGTA, 2 mM HEPES; pH 7.2) containing 4 mM ADP, 2 µM rotenone, 5 mM succinate, and 5 nM XF PMP. Cells were spun down in Seahorse assay medium and then washed twice with MAS before adding MAS supplemented with ADP, succinate, rotenone, and XF PMP. Cells were incubated at 37 °C for 10 min before initiating the respirometry assay with the permeabilized cells starting in State 3 respiration. Injections resulted in a final concentration of 2 µM oligomycin from Port A, 1 µM FCCP from Port B, and 2 µM antimycin A and rotenone from Port C, and 0.5 mM TMPD and 1mM Asc from Port D. Measurements included three State 3 measurements, three measurements after injection of oligomycin, two FCCP measurements, two antimycin A and rotenone measurements, and three measurements after injection of TMPD/Asc. Respiration measurements were conducted with four technical replicates.

#### 2.3.3. RIFS

Respirometry in previously frozen lymphocytes was measured in MAS. Cells were resuspended and spun down in 20 µL of unsupplemented MAS. The samples were centrifuged at 2100× *g* for 10 min and stopped without a break. The samples were brought to a final volume of 150 µL with MAS supplemented with 10 µg/mL cytochrome c and 2.5 µg/mL alamethicin. Injections resulted in a final concentration of either 2 µM rotenone and 5 mM succinate or 1 mM NADH from Port A, 2 µM antimycin A and rotenone from Port B, 0.5 mM TMPD and 1mM Asc from Port C, and 50 mM sodium azide from Port D. Measurements included one measurement before injection of substrates (NADH or succinate and rotenone), two maximal respiration measurements after injection of substrates, two measurements after injection of antimycin A and rotenone, two TMPD/Asc measurements, and two measurements after inhibition of CIV with azide. Respiration was measured in triplicate.

#### 2.3.4. Complex V ATP Hydrolysis Assay

ATP hydrolysis was measured in frozen samples in MAS. Cells were resuspended and spun down in unsupplemented MAS (20 µL of cell suspension). After spinning the cells in the XF96 plate 130 µL MAS containing 5 mM succinate plus 2 µM rotenone was added per well. Injections resulted in a final concentration of 2 µM antimycin A from Port A, 10 mM ATP plus 1 µM FCCP from Port B and C. Port B and C are injected consecutively to assess maximal ATP concentration. Measurements included two measurements before injection of Port A, two measurements after injection of Port A and three maximal ATP hydrolytic activity (ECAR) after injection of Port B/C. Respiration was measured in triplicate.

### 2.4. MitoTracker Deep Red in Frozen Cells

Cells were stained with 500 nM MTDR in PBS for 10 min in a black 96-well plate imaging plate. Cells were then washed with PBS to remove the MTDR. Fluorescence intensity was measured from the bottom of the plate in a Tecan Spark 10 M Multimode Plate Reader using an excitation wavelength of 633 nm and an emission wavelength of 678 nm.

### 2.5. Enzymatic Assays in Frozen Cells

#### 2.5.1. Complex I Activity

CI activity was measured in cell lysates following NADH oxidation at 340 nm as described previously [25].

#### 2.5.2. Citrate Synthase Activity

Citrate synthase activity was performed in cell lysates using DTNB, as described in [5].

### 2.6. Statistical Analysis

Assays were run as proof of concept that the RIFS protocol is comparable to traditional CI spectrophotometric assays and only run in one independent experiment. Therefore, statistics were not performed on the data.

## 3. Gold Standards: Human Samples for Assessment of Mitochondrial Bioenergetic Function

### 3.1. Bioenergetics Testing in Mitochondrial Disorders

Impaired oxidative phosphorylation is a hallmark of mitochondrial disorders with progressive clinical manifestations that can vary from single organ to multisystem disorders, often affecting organs with high energetic demands [26]. Mitochondrial diseases have an estimated prevalence of one in 4300 adults, making them the most common inherited metabolic disorder [27,28,29,30]. Defects in CI are the most commonly observed respiratory chain disorder [31,32,33], making the complex a good candidate for bioenergetic testing.

In patients suspected to have mitochondrial disorders, genetic testing and metabolic screening are recommended before biochemical assays to evaluate mitochondrial function due to the precision in categorizing and less-invasive nature of these measurements. Analyte testing of blood, urine, or even cerebrospinal fluid (CSF) in specific cases, for pyruvate, lactate, amino acids, creatine kinase, and carnitine, can provide information about alterations in metabolism in these patients and provide a biochemical metabolic signature of mitochondrial disease [34,35]. Additionally, pathogenic variants in over 400 genes, both mitochondrial and nuclear, have been identified as contributors to mitochondrial disorders [26]. Pathogenic mutations in mtDNA and nuclear DNA (nDNA) can be used to confirm primary mitochondrial disease due to abnormal OXPHOS function. A genetics-first approach has been facilitated by the availability of exome and whole genome sequencing that has improved the identification of mitochondrial disease genes diagnosis [36,37,38]. Identification of nuclear mitochondrial disease genes are also used to diagnose mitochondrial dysfunctions [39,40,41,42]. Secondary mitochondrial dysfunction, which can be influenced by environmental factors in addition to genetic mutations, adds a further level of complexity and can lead to metabolic and neurodegenerative diseases [43]. While advances in sequencing have made rapid diagnosis possible in a large number of patients, mtDNA or nDNA mutations cannot always be identified even in the presence of a clinical mitochondrial phenotype. Therefore, tissue testing for functional validation may still be necessary [42,44].

Functional, biochemical assays to detect alterations in enzymatic activity, respiration capacity, or ATP synthesis in primary tissue can be used to confirm mitochondrial defects as well as histopathology and mtDNA copy number measurement. However, it is important to note that these assays cannot distinguish between primary defects in OXPHOS enzymes and secondary mitochondrial dysfunctions. Measurement of respirometry and ATP synthesis requires fresh tissue since freezing will disrupt the inner mitochondrial membrane and uncouple oxygen consumption from ATP synthesis. Given the need for same day processing and assaying, the expertise required to run these samples, and limitations in the availability of tissue, bioenergetic alternatives to respirometry and ATP synthesis measurements can be run in frozen samples, such as enzymatic assays measuring ETC complex activities.

Although the electron transport chain and ATP synthesis uncouple when samples are frozen, the mitochondrial electron transport complexes remain structurally intact and functional [45,46]. For that reason, a common method used to assess mitochondrial function in frozen samples has been to measure spectrophotometric enzymatic assays that provide information on the activity of individual ETC complexes or the combination of CI + CIII or CII + CIII. The protocols to measure enzymatic activities often use supraphysiological concentrations of some reagents and non-physiological electron donors and acceptors. Table 3 provides an overview of assays to measure enzymatic activities in mitochondria. Although these measurements were successfully used in a relatively high-throughput manner to diagnose primary mitochondrial diseases, namely diseases caused by a primary defect in electron transport chain function [47,48,49], they cannot provide a single measurement of the coordinated function of the electron transport chain function working at more physiological rates. Taken together, these observations suggest that spectrophotometric assays might be less sensitive to detect defects in mitochondrial architecture and complex ultrastructure, such as the ones associated with secondary mitochondrial dysfunction. However, since the integrated measurement of mitochondrial respiration or ATP synthesis is often unfeasible in these samples these assays provide a useful alternative.

### 3.2. Skeletal Muscle Biopsies

Muscle biopsies have long been the gold standard for investigating changes in mitochondrial function via respirometry in human samples [9,18]. Measurements of mitochondrial function can be performed in intact muscle fibers, permeabilized muscle fibers, or isolated mitochondria from muscle biopsies [13,18]. Skeletal muscle biopsies provide a terminally differentiated, post-mitotic sample that requires less mtDNA replication and may maintain stable levels of mtDNA heteroplasmy compared with mitotic cells [72,73,74]. Skeletal muscles have both high energy demand and mitochondrial content making skeletal muscle a valuable primary tissue sample to measure mitochondrial respiration and ATP synthesis. Measuring respiration in muscle fibers offers the ability to probe specific complexes of the ETC, providing information about underlying mechanisms of altered mitochondrial function. As mentioned previously, mitochondrial bioenergetics can also be assessed in these samples through spectrophotometric assays to measure different metabolic pathways (Table 3) and, in the case of citrate synthase, provide a measure of mitochondrial content. Histology can provide information about mitochondrial structure and bioenergetics through commonly used stains such as modified Gomori trichrome (muscle structure, mitochondrial accumulation, and ragged red fiber), cytochrome oxidase (CIV), succinate dehydrogenase (CII, nDNA encoded), and NADH dehydrogenase (CI), as well as stains to assess intramuscular lipid accumulation [75].

While a wealth of mitochondrial information can be gained from skeletal muscle, this technique can be invasive and not available outside clinical settings or the amount of material obtained can be limiting depending on the technique used [75,76]. For example, open biopsies that require a clinical setting are invasive but provided plenty of material while needle biopsies are less invasive, but the amount of sample obtained may be limiting. While a needle biopsy does not require an operative setting, it does require local anesthesia, sterile conditions, and some specialization to obtain the sample [75,77]. Therefore, there has been a push towards the use of more accessible samples with the development of new techniques. To that point, in vivo, non-invasive applications to measure mitochondrial respiratory capacity are available, such as near-infrared spectroscopy (NIRS), which corresponds with the high-resolution respirometry in muscle biopsies [78]. While the use of this and other in vivo techniques is suitable to study human mitochondrial function, there is still a need for more specific, higher throughput techniques that require less expertise to run for use in translational research studies and clinical applications.

While testing of muscle biopsies was considered the gold standard, before the genomics era, to measure mitochondrial bioenergetic function and diagnosis of primary mitochondrial disease [79], alternative methods would be preferentially useful for diagnosis and research when genetic screening is not enough for categorization of the deficiency [80]. Identifying suitable alternatives to muscle biopsies and systemic biomarkers of mitochondrial function has become a major research focus. This is due to the fact the evaluation of mitochondrial bioenergetic function remains an important parameter for basic research and, in some cases, diagnosis, as a way to determine the extent of mitochondrial dysfunction. Functional readouts of mitochondrial bioenergetics also have applications for translation research and for clinical trials where genetic monitoring may not provide a suitable alternative.

### 3.3. Human Fibroblasts

Fibroblasts from human patients can be generated from minimally invasive samples (1 mm punch skin biopsies) and maintain DNA mutations, although heteroplasmy is not necessarily maintained with passages, and cumulative cellular damage [81]. Respirometry measurements are straightforward in fibroblasts across multiple platforms [82,83,84]. Table 4 provides a general overview of the advantages and limitations of measuring mitochondrial bioenergetics in intact cells, permeabilized cells, and isolated mitochondria, all of which can be utilized in fibroblasts.

Fibroblast cell respirometry has been reported to be a faster and more sensitive measure of electron transport chain defects than traditional spectrophotometry enzymatic assays [85]. In a study of Leigh and Leigh-Like Syndrome patients, respirometry was able to detect impairment in the mitochondrial respiratory chain in 50% of patients that could not be identified with measuring individual ETC complex activities alone [84]. This study demonstrates that detection of causative mutations, biochemical, and respiratory defects vary between individuals and that using combinations of these approaches has the best diagnostic rates. Additionally, enzymatic impairment is not always consistent between muscle biopsies and fibroblasts, suggesting that testing multiple samples, when possible, is the best practice.

Patient fibroblasts provide an important research tool to measure mitochondrial bioenergetics as well as a full characterization of mitochondria including, but not limited to, mitochondrial morphology, turnover, and in-depth analysis of mitochondrial metabolism [86,87]. For research purposes, commercially available fibroblasts can be obtained for different mitochondrial disorders and diseases of aging. This makes it possible to better understand the full range of mitochondrial impairment caused by certain mutations or in particular diseases. It also opens up the ability for pre-clinical assessment of mitochondrial function in these cells in response to interventions, such as compound treatments. Fibroblasts can present the metabolic signatures observed in mitochondrial disease patients, such as a metabolic shift from oxidative phosphorylation to increased glycolysis for ATP synthesis [88,89]. Therefore, these cells can be a particularly important tool to test compounds that are not expected to improve mitochondrial function in healthy, control cells but only under mitochondrial dysfunction. Compound testing in fibroblasts can facilitate determining compound effects on metabolically stressed cells without needing to use exogenous stressors such as calcium, reactive oxygen species (ROS), or mitochondrial inhibitors that can be difficult to titrate and often have narrow exposure windows between impaired function and apoptosis.

#### Considerations for Utilizing Fibroblasts for Respirometry Studies

Given that fibroblasts proliferate in culture, the amount of sample is not limited to the same extent as with tissue biopsies. However, some considerations arise from measuring metabolism in cultured fibroblasts. In vitro culture conditions, such as the amount of glucose in the culture medium, have been shown to change cellular metabolism across multiple cell lines including fibroblasts [90,91,92,93,94]. Costa et al. demonstrate that culturing fibroblasts in low glucose conditions, a more physiological condition than standard high glucose conditions, results in remodeling of the mitochondrial network and a push towards a more oxidative phenotype without causing an extensive metabolic reconfiguration of substrate preference compared with high glucose cultured cells [95]. On the other hand, growing cells in galactose cause both mitochondrial and metabolic remodeling resulting in increased oxygen consumption rates, ATP levels, and mitochondrial biogenesis. Galactose culturing is a useful approach for screening for drug-induced mitochondrial toxicity as it increases sensitivity to mitochondrial respiratory chain inhibitors [96]. Galactose culturing can also improve the testing of interventions that increase mitochondrial bioenergetic function by preventing compensatory ATP synthesis through glycolysis [97]. There are additional considerations besides media composition, for example, modifying the culturing oxygen concentration has been shown to increase the in vitro lifespan of fibroblasts [98]. Therefore, it is important to consider the conditions under which fibroblasts are grown and how these conditions could affect mitochondrial function and metabolism when investigating mitochondrial phenotypes in vitro.

Furthermore, when utilizing primary fibroblasts it is important to consider population doubling time as these cells have limited proliferative capacity and will become senescent over time [99]. Indeed, human skin fibroblasts display hallmarks of aging senescence and are used as a model of aging in vitro [100,101,102]. However, changes in fibroblast metabolism with increasing passage numbers have technical implications for the experimental use of these cells for the investigation of mitochondrial function. Glucose uptake and lactate production increase with fibroblast population doublings, which is consistent with a switch towards glycolysis as cells become senescent [103,104]. Senescent fibroblasts also display changes in mitochondrial bioenergetics with increased mitochondrial membrane potential heterogeneity, a decreased respiratory control ratio, and uncoupling of mitochondrial respiration from ATP synthesis without changes in total respiratory capacity [105]. There is also evidence that mitochondrial content increases in senescent fibroblasts [100]. However, this may represent compensation for increased cell volume that occurs during senescence and not necessarily a change in mitochondrial density [105]. Subculturing of fibroblasts can also induce shifts in mitochondrial heteroplasmy in cells with mtDNA mutations [106]. Taken together, these data highlight the importance of considering passage doubling when using fibroblasts as a model for studying mitochondrial function and comparing across similar passage numbers.

Primary fibroblasts represent the biological and chronological age of the subjects from which they are derived [81,107]. Fibroblasts derived from old subjects display slower proliferation rates and alterations in mitochondrial function and metabolism compared with fibroblasts from young subjects [107,108,109]. Therefore, a final consideration for using fibroblasts for investigating mitochondrial function is what control should be used for patient cells including considerations of age, sex, and genetics. Age and sex-matched fibroblasts should be used as controls and, whenever possible, those cells should be derived from a relative, such as an unaffected sibling, to better control for genetic differences between the patient and control fibroblasts. Another option is to generate isogenic controls using the corrected disease-causing mutation, for example by clustered regularly interspaced short palindromic repeats (CRISPR)—associated protein 9 (CRISPR-CAS9) correction or overexpressing the wild-type complementary DNA (cDNA), making the comparison more precise by keeping the same genetic background [110].

### 3.4. Summary of Benchmark Human Samples to Study Mitochondrial Bioenergetics

There is growing evidence that impaired mitochondrial function contributes to aging and age-related diseases. Age-related impairments in mitochondrial function include decreased OXPHOS capacity and ATP synthesis and well as increased ROS generation and accumulation of mtDNA mutations and deletions [111,112,113]. Therefore, monitoring mitochondrial function could provide a useful clinical tool for both diagnosis and monitoring the effectiveness of treatments. To accomplish this, there is a need for a less invasive way to monitor mitochondrial function than testing primary tissues. While fibroblasts provide an important research model that can be used for disease diagnosis and pre-clinical research, this approach is not suitable for clinical testing and monitoring due to poor scalability. Muscle biopsies are optimal for bioenergetics measurements, nevertheless collecting these samples is often considered as a last resort due to availability and invasiveness [42]. Therefore, other minimally invasive samples are needed to provide a translational approach to monitoring mitochondrial respiratory function. There is growing evidence that respirometry measurements in circulating blood cells may be a systemic biomarker of mitochondrial bioenergetic function. Blood-based bioenergetics present a model that is minimally invasive to obtain and more feasible to test in clinical settings and on a larger scale in clinical trials.

## 4. Blood-Based Bioenergetics—Respirometry as a Systemic Biomarker of Mitochondrial Function

Mitochondrial content, substrate preference, protein composition, and mitochondrial morphology vary between tissues based on energetic and metabolic demands. Therefore, there are clear advantages to measuring mitochondrial function in the tissue of interest, which is the approach commonly used in basic research. However, primary tissue samples can be difficult to obtain in human subjects and the amount of sample can be limiting for downstream bioenergetic applications. Therefore, functional measurements of mitochondria cannot always be used in translational research or to identify impairment in mitochondrial function clinically, even if it would be a useful readout. While there is differential mitochondrial function between tissues, there is also evidence that circulating cells can act as a biomarker for overall mitochondrial function. Using platelets and leukocytes as a less invasive biomarker of mitochondrial function has emerged as a potential approach for situations when testing primary tissue samples is not feasible. These cells represent an attractive systemic biomarker candidate and an alternative to invasive tissue biopsies in human studies, since blood can be easily obtained in sufficient quantities for respirometry assays and other bioenergetic measurements. Additionally, isolated blood cells can be cryopreserved for later testing if the cells need to be isolated at an off-site location and shipped for respirometry analysis. Cryopreservation allows for samples that are collected at different times to be stored and subsequently run together in the same experiment, which may cut down on variability. However, long-term cryopreservation can also decrease cell viability and impair mitochondrial bioenergetic parameters and should be considered when using this approach [114].

Protocols for measuring respirometry in circulating blood cells have been well established for both intact and permeabilized cells and previously described [115,116,117]. Oxygen consumption measurements in circulating blood cells are compatible with any respirometry platform. Given that platelets and leukocytes are non-adherent, they are well suited for measurements in oxygraph closed-chamber respirometers, but they can also be tested in plate-based respirometers by coating plates to promote cell adhesion. The primary advantage of using a plate-based respirometer, such as the Seahorse Extracellular Flux Analyzer, is minimizing the amount of sample required for measurements, which is about ten times less in the Seahorse XF96 compared with an oxygraph [118].

Tyrrell et al. have compared respirometry in permeabilized muscle fibers to intact platelets and monocytes in healthy vervet/African green monkeys [119]. Their data demonstrate that the maximal respiratory capacity of circulating blood cells correlates with the maximal OXPHOS capacity of permeabilized skeletal muscle from the same animal. Both platelet and monocyte maximal oxygen consumption displayed a significant correlation with (1) the combined CI and CII-linked OXPHOS (State 3 respiration) in permeabilized skeletal muscle fibers and (2) the respiratory control ratio in isolated skeletal muscle mitochondria. Comparisons of skeletal muscle bioenergetics and circulating blood cells have also been completed in human subjects [120]. Braganza et al. show significant correlations between bioenergetic parameters in platelets and permeabilized skeletal muscle fibers from the same subject. Maximal respiration in platelets significantly correlated with maximal respiration in muscle and platelet proton leak correlated with State 4 (leak) respiration in muscle. Furthermore, basal and ATP-linked respiration was decreased in older subjects compared with a younger cohort. However, not all studies demonstrate correlations between circulating blood cells and muscle fibers. In a study of 32 women, Rose et al. did not observe a correlation in intact respirometry in peripheral blood mononuclear cells (PBMCs) or platelets with permeabilized muscle fibers, though there were some correlations with permeabilized platelets and permeabilized muscle [118]. Taken together, these results suggest that bioenergetic profiling of platelets and leukocytes may be used as a biomarker of mitochondrial function and, in some cases, reflect the bioenergetic capacity of muscle tissues and potentially other highly metabolic primary tissues.

Pecina et al. could identify defects in CI in permeabilized lymphocytes isolated from pediatric patients, some with known mitochondrial disorders, using high-resolution respirometry [121]. The authors used a combined approach testing functional methods and protein analysis and were able to detect several types of isolated OXPHOS disorders. They suggest that lymphocytes are a suitable sample to detect isolated defects of CI, CIV, and ATP synthase and that repeated analysis of lymphocytes could allow for monitoring changes in mitochondrial function during disease progression. Additionally, changes in platelet and leukocyte mitochondrial respiratory function have been reported in several diseases including type 2 diabetes, human immunodeficiency virus (HIV), neurodegenerative diseases, as well as in aging [120,122,123,124,125,126,127,128,129,130,131,132]. Interestingly, there does not seem to be a single respiratory parameter that demonstrates consistent differences in healthy controls compared with disease patients. Changes in respiration, both increased and decreased, have been found in basal, ATP-linked, and maximal respiration [133], can be variable, and sex-associated differences in oxygen consumption rates have been reported [130,134]. The wide range of mitochondrial changes observed in these studies could be due to different bioenergetic impairments and compensations between diseases. Additionally, there could be differences arising from the subpopulation of cells tested or potential confounding factors such as the presence of other, contaminating cell types, or inadvertent cell activation during isolation.

### Considerations for Utilizing Circulating Blood Cells for Respirometry Studies

An important consideration for blood-based bioenergetics is selecting an appropriate cell type to use for respirometry experiments and whether mixed cell populations, such as PBMCs, are suitable samples for respirometry. Rausser et al. used a high-throughput mitochondrial phenotyping platform to compare PBMCs to leukocyte subtypes from the same individual [135]. They show large functional differences in both mitochondrial content and respiratory chain enzymatic activity between immune cell subtypes. Furthermore, PBMC cell type composition can vary between individuals and within the same individual over time. Therefore, they caution that using PBMCs for mitochondrial bioenergetics measurements can mask age- and sex-related changes. Furthermore, Darley-Usmar et al. have shown that respiration rates, the ratio of OXPHOS to glycolysis, as well as mitochondrial protein composition vary between circulating blood cell subpopulations [116]. For example, platelets have the highest basal and ATP-linked OCR compared with leukocyte cell types but lower maximal respiration and reserve capacity [116,136], which could alter bioenergetic results and interpretation if platelet contamination is present in the test samples.

Platelets are a common contaminant in human PBMC samples and vary depending on blood collection and PBMC isolation strategies [137]. Urata et al. have demonstrated that platelet contamination is common and can vary to a large extent amongst PBMC preparations [138]. They measured mtDNA copy number before and after platelet depletion and showed that the amount of platelet contamination varied between individuals and that PBMC mtDNA content is overestimated almost two-fold due to platelet contamination. This work was framed in the context of using PBMC mtDNA copy number as a potential biomarker to monitor disease but has implications for blood-based bioenergetics, as well. Additionally, cellular and mitochondrial metabolism including mitochondrial bioenergetic profiles changes in resting cells compared with activated cells [122,139]. Thrombin-activated platelets increase basal and ATP-linked oxygen consumption and glycolysis compared with control cells [140]. Therefore, it is important to consider cell activation, optimal collection, and isolation conditions when isolating platelets and leukocytes from whole blood [141,142].

For further information on respirometry in circulating blood cells, Braganza et al. have published a comprehensive review of blood-based bioenergetics outlining the research published in platelets and leukocytes as well as considerations for the use of blood-based bioenergetics as a translational and clinical tool [133]. While more research is needed to understand what cell populations are most representative of systemic mitochondrial function and specific diseases, blood-based bioenergetics offers a minimally invasive alternative to muscle biopsies and could provide a systemic biomarker of mitochondrial function and key readout for clinical trials. However, one major consideration for the utilization of this approach is that samples generally need to be collected and immediately processed to isolate circulating platelets and leukocytes from whole blood. Further isolating subpopulations of cells requires more starting material and processing. Therefore, there is still a need for a non-invasive sample that requires minimal on-site processing.

## 5. Mitochondrial Function in Previously Frozen Specimens: Overview and Commentary on an Updated Approach to Respirometry

Several attempts have been made to carry out respirometry on cryopreserved tissue samples. In these approaches, samples were collected and frozen in different solutions trying to preserve the structure and integrity of the mitochondrial membrane. However, the outcome of these attempts was variable and not standardized [143,144,145,146,147]. Mitochondrial complexes and supercomplexes can be isolated from frozen samples and separated in native gels. These isolated complexes and supercomplexes preserve their enzymatic activity and more interestingly, mitochondrial supercomplexes respire [45,46]. These works demonstrate that the electron transport chain is not destroyed by freeze-thawing and that previously frozen sample respirometry is feasible [46]. The challenge is to convert the assay performed in isolated mitochondrial supercomplexes to previously frozen samples, without the need to isolate the ETC components.

We have recently published a new approach to measure respirometry in frozen samples (RIFS) that reconstitutes maximal mitochondrial respiration in previously frozen samples [148,149]. Using RIFS, maximal oxygen consumption of the ETC is measured by providing physiological electron donors and acceptors with oxygen consumption as an integrated readout. It is important to consider that in previously frozen samples, the natural provider of electron equivalents, the tricarboxylic acid (TCA) cycle, is not directly feeding the ETC. For that reason, we need to bypass the components that are lost during the freeze-thaw step by providing electron donors directly to the ETC and correct for variable permeabilization of mitochondrial membranes, when necessary. For example, NADH is used rather than pyruvate + malate since NADH, a reducing equivalent that donates electrons to CI of the ETC, does not have a permeability barrier in frozen tissue. The protocol is straightforward in almost all cases except for tissues that contain a high proportion of fibers and collagen such as skeletal muscle or tissues that are rich in membranes such as the brain. In these tissues, an additional protocol step is required for either enzymatic digestion (skeletal muscle) or additional membrane permeabilization (brain) for the reagents and electron donors, particularly NADH, to be accessible to mitochondria.

**Figure 2:**
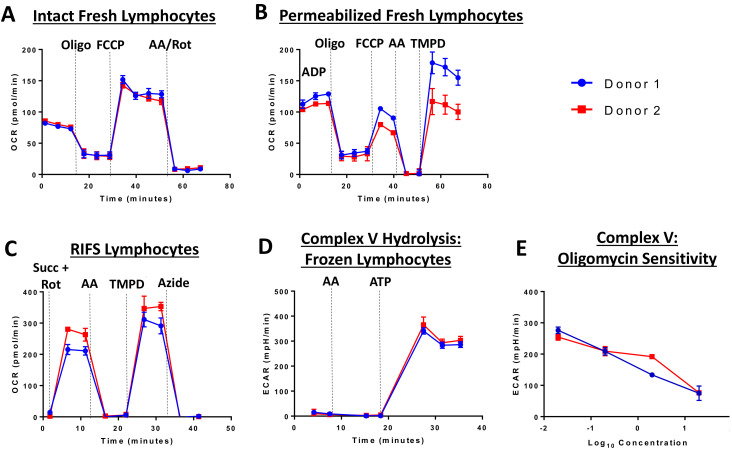
Extracellular flux analysis in fresh and frozen lymphocytes from two donors. Respirometry was measured in fresh (**A**,**B**) and frozen lymphocytes (**C**–**E**). (**A**) Oxygen consumption was measured in intact cells before and after injection of oligomycin (CV inhibitor), FCCP (chemical uncoupler), and antimycin A (CIII inhibitor) and rotenone (CI inhibitor). (**B**) Respiration in permeabilized cells with CII substrates. A final injection of TMPD was included to measure CIV. (**C**) RIFS protocol with succinate and rotenone to drive respiration through CII. (**D**) CV activity measured by media acidification as a result of ATP hydrolysis. (**E**) Oligomycin-sensitivity in the CV measurement. Abbreviations: AA: Antimycin A; ATP: Adenosine triphosphate; FCCP: Carbonyl cyanide p-trifluoromethoxyphenylhydrazone; oligo: Oligomycin; Rot: Rotenone; Succ: Succinate; TMPD: N, N, N’, N’-tetramethyl-p-phenylenediamine.

The RIFS protocol was optimized and validated using the Seahorse XF96 Extracellular Flux Analyzer, but should be suitable for any respirometry platform. The RIFS assay includes injection of NADH to measure maximal CI respiratory capacity, succinate to measure maximal CII respiratory capacity, as well as TMPD to measure CIV activity. TMPD provides electrons to CIV by directly reducing cytochrome c. Inhibitors of CI, CIII, and CIV are used as controls in each assay to determine sensitivity to respiratory chain complexes. RIFS preserves 90–95% of the maximal respiratory capacity in frozen samples and can be applied to isolated mitochondria, cells, and tissue homogenates with high sensitivity. As an example in cells, Figure 2 compares fresh and frozen respiration in human lymphocytes from two donors. In fresh intact and permeabilized cells, these lymphocyte samples demonstrated low inter-individual variability and the expected response to injected compounds in all assays (Figure 2A,B). However, oxygen consumption rates were lower than expected in permeabilized cells possibly due to incomplete permeabilization. RIFS formulation allows for simultaneous measurements of respiration driven by electron entry through CI and CII while determining individual CIV activity. Additionally, CV, or ATP synthase, activity can be measured via ATP hydrolysis. In lymphocyte cell lysates, respiration driven through CI and CII, as well as CIV and CV activity can be measured with low inter-individual variability. Furthermore, CV is sensitive to dose-dependent inhibition with oligomycin (Figure 2C–E).

To measure CV activity, the extracellular acidification (ECAR) is measured to monitor the acidification of the experimental medium in response to hydrolysis of injected, saturating levels of ATP, as previously described in Divakaruni et al. [150]. CV activity can be measured in previously isolated, frozen mitochondria as well as tissue and cell homogenates. In this application, the addition of the CV inhibitor oligomycin acts as an important control to determine that the increased ECAR after injection of ATP is a result of ATP hydrolysis and not non-specific acidification.

This ECAR approach will provide a measure of maximal hydrolytic capacity as a readout of CV enzymatic activity. However, it is important to note that ATP hydrolysis, both through ECAR measurements or with spectrophotometric assays, may underestimate CV deficiencies depending on the specific mutated subunit. For example, mutations in the mtDNA-encoded ATP synthase F_o_ subunit 6 that cause severe impairment in ATP synthesis may show little or no defects in ATP hydrolysis [151]. Therefore, while CV activity is a useful readout, it cannot replace measurement of ATP synthesis or respirometry in fresh samples or provide information on physiological levels of ATP hydrolysis.

The simplified sample preparation with RIFS, particularly with homogenates, and the 96-well format of the Seahorse XF96 Analyzer allows using significantly less biological material. RIFS does not require isolation of mitochondria or the use of detergents or plasma membrane permeabilizers, which simplifies the methodology and minimizes changes induced by partial or over permeabilization due to the use of detergents. Acin-Perez et al. provide validation in mouse, zebrafish, and human samples [148]. Another advantage of using total tissue lysates is that it accounts for tissue-specific mitochondrial function, where, for example, soleus showed significantly higher respiration than quadriceps, as previously described [152]. In homogenates and cell lysates, respirometry can also be normalized to mitochondrial content to provide a measure of respiration per functional unit. In addition to traditional methods to measure mitochondrial mass, we have recently described a high throughput MitoTracker Deep Red protocol that requires minimal biological samples that can be run in parallel to RIFS and used to normalize respiration [148,149]. Since differences in mitochondrial content can account for differences in respiration rates in homogenates, the normalization step can differentiate between changes in mitochondrial content and function when homogenate samples or cell lysates are loaded per total protein. Normalization to mitochondrial content is not necessary with isolated mitochondria since respiration in these samples is already normalized to mitochondrial content when loaded per total protein.

**Figure 3:**
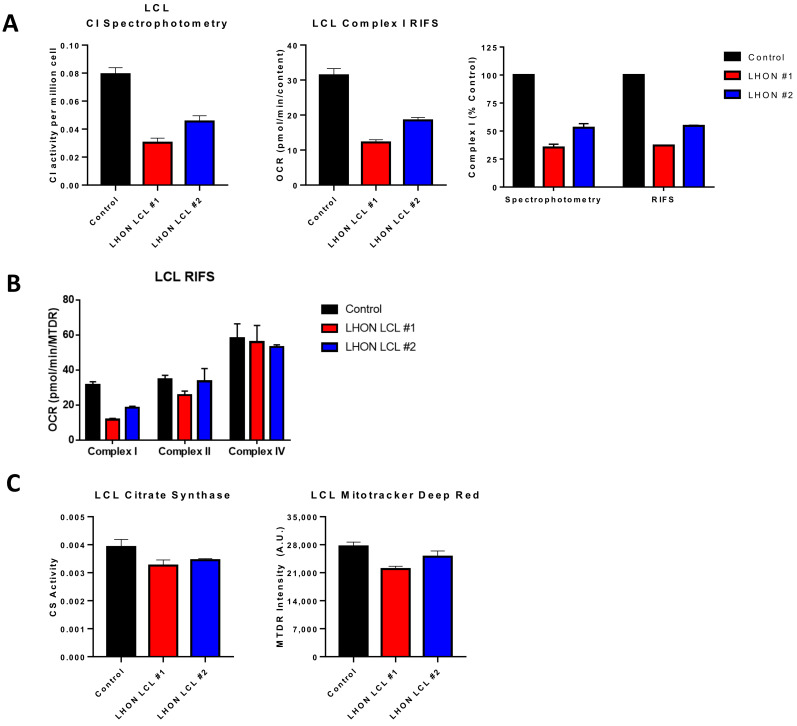
Comparison of RIFS and spectrophotometric approaches in Leber optic atrophy lymphoblastoid cell lines. (**A**) CI activity compared with the RIFS protocol. The spectrophotometric CI assay required 0.5 × 10^6^ cells/well compared with 0.2 × 10^6^ cells/well with RIFS. The data were also normalized to control cells as a more direct comparison of the two techniques. (**B**) CI, CII, and CIV results were obtained using RIFS. (**C**) Mitochondrial content measurements with citrate synthase activity and the MitoTracker Deep Red plate reader assay. Abbreviations: LCL: Lymphoblastoid cell line; LHON: Leber optic atrophy; RIFS: Respirometry in frozen samples.

**Figure 4:**
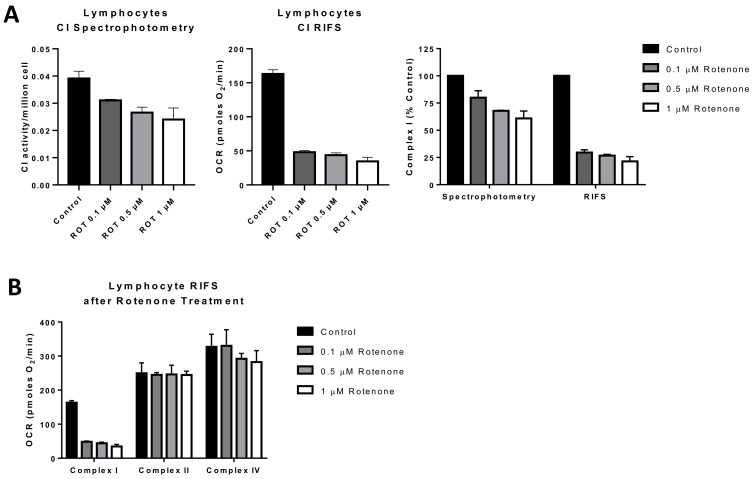
Comparison of RIFS and spectrophotometric approaches to measure Complex I in human lymphocytes treated with rotenone prior to freezing. (**A**) CI activity compared with the RIFS protocol. The spectrophotometric CI assay required 1 × 10^6^ cells/well compared with 0.4 × 10^6^ cells/well with RIFS. The data were also normalized to control cells as a more direct comparison of the two techniques. (**B**) Compiled CI, CII, and CIV results from two donors were obtained using RIFS. Abbreviations: CI: Complex I; RIFS: Respirometry in frozen samples; ROT: Rotenone.

The RIFS technique demonstrated that highly oxidative tissues such as heart, brown adipose tissue, and brain showed higher mitochondrial respiration rates per milligram of tissue whereas white adipose tissue, known for its low mitochondrial content, showed the lowest respiration rates. These results suggest that RIFS reveals physiological differences depending on the energy and metabolic demand of the tissue. When comparing RIFS to traditional methods used in frozen samples such as spectrophotometric enzymatic assays, the latter provides information about the maximal activity of the individual complexes or some of them combined, whereas RIFS allows for an integrative measure of the ETC from CI or CII to the natural electron acceptor that is oxygen. Additionally, RIFS often requires less sample to perform the assay compared to enzymatic assays and can show increased sensitivity to specific inhibition of the ETC complexes, as displayed in Figure 3 and Figure 4 where results were obtained with half as much starting material compared with enzymatic assays. In addition, the assay is straightforward where the number of reagents needed is minimal in comparison to spectrophotometric assays. The simple, standardized protocol of RIFS could limit the inter-laboratory variability that has been reported with enzymatic spectrophotometric assays [153].

Snap freezing samples in liquid nitrogen is the best approach for RIFS. However, due to possible delays between sample collection, freezing, and storage for clinical samples, an initial concern was how quickly samples need to be frozen to preserve mitochondrial function. For that reason, different sampling procedures were tested to determine which approach best preserved maximal respiratory rates when samples could not be immediately flash-frozen in liquid nitrogen. Samples placed on ice for up to 3 h after collection as well as samples immediately stored at −20 °C preserved the integrity and function of mitochondria ETC complexes suggesting that this approach could be suitable for samples that require a delay in processing [148,149].

### 5.1. Applications for Respirometry in Previously Frozen Samples

There are numerous applications where RIFS can be used in preexisting, stored frozen samples. In adipose samples from pheochromocytoma patients, differences in maximal respiratory capacity that were observed in fresh clinical samples were maintained after long-term storage at −80 °C [154]. RIFS generated comparable data to those initially obtained in freshly isolated mitochondria including increased respiration through CI, CII, and CIV and increased mitochondrial content. Furthermore, RIFS made it possible to assay all the samples together compared to fresh samples that had to be run one at a time. This application could be particularly important in age-related studies since it enables a longitudinal design in which bioenergetic profiling can be monitored and compared over years across the same patients. RIFS could also be used as a functional test for patients with mitochondrial disease using less invasive samples. We tested the RIFS protocol in lymphoblastoid cell lines (LCLs), an immortalized B lymphocyte cell line, from patients with the mitochondrial disorder Leber optic atrophy (LHON). These cells were derived from patients with a mtDNA mutation in the NADH dehydrogenase subunit 4 gene. Both RIFS and spectrophotometric assays were able to detect impaired CI activity compared with control cells and revealed similar differences between the LHON lines (Figure 3A). Moreover, we were able to detect impaired NADH-driven respiration with half the amount of sample or the same amount of sample while simultaneously getting information about CII-driven respiration and CIV activity, both of which were, for the most part, similar to the control cells with the exception of LHON LCL line 1 that showed some decrease in maximal CII respiration (Figure 3B). Comparing a citrate synthase assay to the MTDR assay to measure mitochondrial content, the results from both assays were comparable, but again RIFS required less starting material, fewer reagents, and was less time-sensitive than the citrate synthase assay (Figure 3C).

An additional potential application for RIFS is in monitoring environmental toxicants to which mitochondria are a key target [155]. For that reason, applications targeted to direct screening of compounds leading to mitochondrial toxicity can benefit from RIFS by testing the functional impact of environmental exposures within a large population [155,156]. We tested rotenone exposure in lymphocytes treated with different concentrations of rotenone before freezing and compared the RIFS protocol to a CI activity spectrophotometric assay. Both approaches were able to reveal impairment in CI, but the RIFS assay was more than twice as sensitive and required half as much starting material (Figure 4A). Additionally, given the high oxygen consumption rates with the RIFS protocol, it is likely that less material is required than was used in these proof-of-concept assays (0.4 × 10^6^ cells/well). The requirement of less starting material could be particularly important when the amount of sample is limited. In addition to CI, RIFS also provide information on CII and CIV demonstrating that the rotenone-induced defect was limited to CI (Figure 4B).

### 5.2. Limitations of RIFS

While the RIFS protocol was not developed to replace traditional respirometry approaches, it is an alternative method when using fresh samples is not feasible due to sample size and availability, or immediate access to instrumentation is not possible, a problem particularly relevant for testing human samples. Table 5 provides a comparison of the advantages and disadvantages of measuring respiration in fresh and frozen tissue samples. Respirometry in fresh samples will undoubtedly provide the most complete information about mitochondrial function and the bioenergetics of a sample given the coupled nature of fresh samples, that is, respiration is coupled to ATP synthesis. However, RIFS can provide a convenient alternative when respiration in fresh samples is not feasible or as a complementary method to determine maximal respiratory capacity in an integrated, yet uncoupled, system.

Since freeze-thaw cycles will disrupt the integrity of the mitochondrial membranes and results in the loss of soluble matrix components, an important limitation is that coupled respiration or ATP synthesis cannot be measured with RIFS. These limitations should be considered when using this approach as it will not be sensitive to changes in substrate transport, substrate oxidation, ATP turnover, or TCA cycle flux. However, it has been demonstrated that disease-related phenotype often correlates with a decrease in maximal respiration [119,157,158,159] and that defects in coupled respiration are mostly related to some mechanism of uncommon drug toxicity [160]. Therefore, there are multiple applications for RIFS when testing fresh samples is logistically impracticable. While the data presented in this review are preliminary as proof of concepts for use of RIFS in circulating blood cells, the data suggest it could complement the current blood-based bioenergetics approaches. Furthermore, as the field continues to search for a biomarker of mitochondrial function, the RIFS approach provides an additional method for measuring maximal mitochondrial respiration and may be useful for non-invasive samples.

## 6. Future Outlook: Respirometry in Non-Invasive Samples

In the last decade, there have been substantial improvements in approaches to measure mitochondrial function in human samples. In particular, measurement of mitochondrial function in circulating platelets and leukocytes has elevated translational mitochondrial research and improved the prospects of clinical monitoring of mitochondrial bioenergetics. However, blood collection still needs to be completed in a medical setting and requires fresh processing. This processing can be intensive, especially when isolating sub-populations of PBMCs, which can present challenges in the ability to scale up for clinical trials. Additionally, blood-based bioenergetics do not always demonstrate changes in mitochondrial function observed in primary tissues [161], which suggests that subpopulation testing is an important consideration as subpopulation bioenergetic correlations may differ between tissues and diseases. More research is needed to understand the bioenergetic differences between subpopulations, population variability, and selection of the appropriate cell type so that this approach can be standardized. With the growing number of mitochondrial drug targets being developed and tested in clinical trials, there is a need for further minimally/non-invasive samples that can be scaled up for clinical testing and large-scale population studies. The RIFS protocol has the potential to be used for these applications if it can be optimized from non-invasive samples such as cells from urine or saliva, or buccal mucosa cells collected from cheek swabs, which can be easily obtained and immediately frozen without processing for later testing. Since RIFS requires minimal starting material, it could be modified for use in non-invasive samples that require minimal processing. While it is still unknown whether RIFS will be a suitable approach for non-invasive samples, there is some evidence that buccal swabs may be an interesting foundation for the development of non-invasive samples.

Buccal mucosa cells represent a potential non-invasive sample that can be used for mitochondrial bioenergetics profiling. Buccal swab testing of electron transport chain activity is possible [162], but has been reported to show poor sensitivity and specificity for mitochondrial disease [41]. Frederiksen et al. showed that there is a significant correlation in mutation load of the common A3243G mtDNA point mutation in four different tissues: blood leucocytes, buccal cells, skeletal muscle cells, and urine epithelial cells [163]. They suggest that genetic testing of buccal cells and urine epithelial cells may provide an important supplement to the diagnosis of mitochondrial diseases, but also note that skeletal muscle was still the most informative tissue with the highest mutation load [163]. Buccal swabs from a 6-year old patient with suspected myoclonic epilepsy and ragged red fibers (MERRF) presented common mtDNA deletions and decreased ETC activities compared with controls [164]. In a study of 40 patients with suspected mitochondrial disease, when comparing enzymatic activities in buccal mucosa cells to muscle biopsies, there was a 77% correlation for an isolated CI deficiency, 86% for combined CI and CIV defects, and 100% in CIV. Overall, detection of mitochondrial impairment was 82% in buccal swabs compared to muscle biopsies [165]. Alterations in buccal mitochondrial DNA and function have also been reported in lung cancer patients and autism spectrum disorder patients (ASD) [166,167]. Additionally, there is evidence that buccal swab samples can be used to monitor mitochondrial enzymatic activity in response to treatment. In a study of ASD children with or without mitochondrial disease, CI, CIV, and citrate synthase activity in response to fatty acid and folate supplementation was measured in buccal extracts [168]. The results suggest that changes in CI and citrate synthase activities can be observed in buccal swabs in response to common interventions and that increased activity in these enzymes was more discernable in the mitochondrial disease subgroup.

While studies measuring mitochondrial function in buccal swabs are still limited, enough data demonstrate mitochondrial functional changes in buccal swabs to warrant further investigation. It is possible that an integrated readout, such as RIFS, would provide both better sensitivity and specificity for buccal samples. However, a potential limitation to buccal swab bioenergetics measurements could be bacterial contamination that decreases measurement specificity to the human samples [169,170]. Removal of bacteria from these samples will likely be an important step to improve sensitivity in the respirometry assay and ensure that outcomes are accurate. While this approach in buccal mucosa is still hypothetical and would require optimization and validation, it could provide a method to rapidly screen maximal mitochondrial respiratory capacity and provide a complementary approach to blood-based bioenergetic measurements and measurements in primary tissues.

## 7. Summary

Impaired mitochondrial function has been shown to contribute to the development and progression of various diseases. There is an unmet need to clinically monitor mitochondrial function. However, until now this has been limited due to the labor-intensive immediate processing of the samples. Blood-based bioenergetics have met some of this need but do not exclude the necessity of immediate processing. With the development of RIFS as a complementary technique to measure mitochondrial function in previously frozen samples, the possibility of clinical monitoring of mitochondrial function in samples collected at remote sites, or retrospectively in samples residing in tissue biobanks becomes more apparent. While these emerging approaches require further optimization, in some cases, and validation of population variability and sensitivity to detect differences between healthy and disease patients, they represent a potential step forward in translation bioenergetic monitoring in human samples.

## Figures and Tables

**Table 1:** Vendor and catalog number information for reagents used in the proof of concept assays completed for this review.

Reagent	Supplier	Catalog Number
Acetyl-CoA	Sigma	A2181
Adenosine 5′Diphosphate (ADP)	Sigma	A5285
ATP	Sigma	A26209
Alamethicin	Sigma	A4665
Antimycin A (AA)	Sigma	A8674
Ascorbic Acid (Asc)	Fisher	A61-100
Azide	Sigma	S8032
Coenzyme Q1	Sigma	C7956
Cytochrome c	Sigma	C2506
Dulbecco’s Modified Eagle Medium (DMEM)	Sigma	D5030
5,5-dithiobis(2-nitrobenzoic acid) (DTNB)	Sigma	D218200
Ethylene glycol-bis(2-aminoethylether)- N,N,N’,N’-tetraacetic acid (EGTA)	Sigma	E4378
FBS	Corning	35010CV
Carbonyl cyanide p-trifluoromethoxyphenylhydrazone (FCCP)	Enzo	BML-CM120
Glucose	Sigma	G5767
Glutamine	Gibco	25030081
4-(2-hydroxyethyl)-1-piperazineethanesulfonic acid (HEPES)	Gibco	15630080
Reduced nicotinamide adenine dinucleotide (NADH)	Sigma	N8129
Magnesium Chloride	Sigma	M0250
Mannitol	Sigma	M9546
MitoTracker Deep Red (MTDR)	Invitrogen	M22426
Oligomycin (Oligo)	Sigma	495455
Oxaloacetic acid	Sigma	O4126
Phosphate Buffered Saline (PBS)	Gibco	14190250
Poly-D-Lysine	Sigma	P6407
Potassium cyanide	Sigma	60178
Potassium phosphate	Sigma	P5655
RPMI-1640	Gibco	11875119
Rotenone (Rot)	Sigma	R8875
Sodium Pyruvate	Gibco	11360070
Succinic Acid (Succ)	Sigma	S9512
Sucrose	Sigma	S0389
N, N, N’, N’-tetramethyl-p-phenylenediamine (TMPD)	Sigma	87890
*XF* plasma membrane permeabilizer (XF PMP)	Agilent	102504-100

**Table 2:** The mitochondrial compounds/substrates used to investigate mitochondrial function in fresh and frozen samples. The compounds used to measure mitochondrial respiratory function and their corresponding functions are listed. Abbreviations: ADP: Adenosine diphosphate; ATP: Adenosine triphosphate; CI: Complex I; CII: Complex II; CIII: Complex III; CIV: Complex IV; CV: Complex V; ETC: Electron transport chain; NADH: Reduced nicotinamide adenine dinucleotide; OXPHOS: Oxidative phosphorylation; TMPD: N, N, N’, N’-tetramethyl-p-phenylenediamine.

Mitochondrial Compound/Substrate	Function
ADP	Induce State 3, ADP-stimulated respiration
ATP	Induce Complex V (CV) hydrolysis in uncoupled mitochondria
Alamethicin	Permeabilize the mitochondrial inner membrane
Antimycin A	Complex III (CIII) inhibitor
Ascorbate	Maintains TMPD in the reduced state
Azide	Complex IV (CIV) inhibitor
Cytochrome c	Soluble component of ETC; added to replace the cytochrome c that is lost with freezing
FCCP	Chemical uncoupler of OXPHOS to measure maximal respiration
NADH	Complex I (CI) substrate
MitoTracker Deep Red	Fluorescent dye to measure mitochondrial content
Oligomycin	CV inhibitor
Rotenone	CI inhibitor
recombinant perfringolysin O (rPFO) or XF PMP	Permeabilize the plasma membrane
Succinate	Complex II (CII) substrate
TMPD	Electron donor to cytochrome c/CIV

**Table 3:** Spectrophotometric approaches to measure mitochondrial enzymatic activities for assessment of mitochondrial bioenergetic function. The table presents the metabolic enzyme of interest, the metabolic pathway the measurement would evaluate, and methodology references. Abbreviations: ATP: Adenosine triphosphate; CI: Complex I; CII: Complex II; CIII: Complex III; CIV: Complex IV; DH: Dehydrogenase; ETC: Electron transport chain; NADH: Reduced nicotinamide adenine dinucleotide; OXPHOS: Oxidative phosphorylation; TCA: Tricarboxylic acid cycle.

Enzyme	Metabolic Pathway	References
CI: NADH/CoQ1 Oxidoreductase	ETC, OXPHOS	[50,51]
CII: Succinate DH	ETC, OXPHOS, TCA	[25,50,51]
CIII: Coenzyme Q: Cytochrome c—oxidoreductase	ETC, OXPHOS	[50,51]
CIV: Cytochrome c oxidoreductase	ETC, OXPHOS	[50,51]
Combined CI + CIII	ETC, OXPHOS	[51]
Combined CII + CIII	ETC, OXPHOS	[51]
Combined CI + CIII + CIV	ETC, OXPHOS	[52]
CV: ATP hydrolysis	ETC, OXPHOS	[53,54]
Creatine kinase	ATP homeostasis	[55]
Adenylate kinase	ATP homeostasis	[55]
Citrate synthase	TCA	[50,51]
α-ketoglutarate DH	TCA	[56,57]
Isocitrate DH	TCA	[58]
Malate DH	TCA	[55,56,57]
Aconitase	TCA, Redox balance	[50,56,59]
Manganese Superoxide dismutase (MnSOD)	Redox balance	[60]
Nicotinamide nucleotide transhydrogenase	Redox balance	[61,62,63]
Catalase	Redox balance	[56]
Glycerol-3 phosphate DH	Glycerophosphate shuttle	[25,51]
Pyruvate DH	Glucose and Fatty acid oxidation	[64]
β-Hydroxyacyl CoA DH	Fatty acid oxidation	[50,55]
Short-chain hydroxyl-acyl-CoA DH	Fatty acid oxidation	[58,65,66]
β-hydroxybutyrate DH	Ketone body	[67,68,69]
Hexokinase	Glucose metabolism	[55,70]
Arginase	Urea cycle	[71]

**Table 4:** Advantages and disadvantages of respirometry measurements in intact and permeabilized cells and isolated mitochondria. Adapted from [12]. Abbreviations: ETC: Electron transport chain; OCR: Oxygen consumption rate.

	Intact	Permeabilized	Isolated Mitochondria
Advantages	Greater physiological context Cellular control of metabolism/nutrient preference Intact mitochondrial architecture Information for oxygen consumption rate (OCR) and glycolysis can be obtained simultaneously Limited material needed	Experimental control of substrates Mechanistic analysis of ETC and mitochondrial metabolism Native intracellular environment Intact mitochondrial architecture Limited material needed	Experimental control of substrates Extra-mitochondrial metabolism does not limit O2 consumption Mechanistic analysis of ETC and mitochondrial metabolism Reproducible, robust OCRs with high dynamic range Normalizing to total protein should normalize for mitochondrial content
Drawbacks	Cell culture conditions, confluence, and experi-mental media influence outcomes ELess mechanistic insight May be more difficult to interpret since so many processes can alter OCR Changes in OCR could reflect changes in mito-chondrial content Normalization is required	No cellular control of metabolism/nutrient pref-erence Careful titration of permeabilizing agent required Changes in OCR could reflect changes in mito-chondrial content Normalization is required Normalization is required	No cellular control of metabolism/nutrient preference Loss of intracellular environment Loss of mitochondrial morphology Isolation procedure can damage mitochondria Selection bias More starting material required
Drawbacks	• Cell culture conditions, confluence, and experimental media influence outcomes • Less mechanistic insight • May be more difficult to interpret since so many processes can alter OCR • Changes in OCR could reflect changes in mitochondrial content • Normalization is required	• No cellular control of metabolism/nutrient preference • Careful titration of permeabilizing agent required • Changes in OCR could reflect changes in mitochondrial content • Normalization is required	• No cellular control of metabolism/nutrient preference • Loss of intracellular environment • Loss of mitochondrial morphology • Isolation procedure can damage mitochondria • Selection bias • More starting material required

**Table 5:** Advantages and disadvantages of respirometry measurements in fresh and frozen tissue samples. Abbreviations: ATP: Adenosine triphosphate; TCA: Tricarboxylic acid cycle.

	Fresh	Frozen
Advantages	Greater physiological context Coupled mitochondria that synthesize ATP Intact mitochondria that maintain morphology with some techniques Metabolic pathways and transporters can be investigated Respiration can be measured in intact and permeabilized cells and isolated mitochondria	Integrated oxygen consumption measurement Maximal respiratory capacity often correlates with disease phenotypes Minimal sample requirement and processing Samples can be collected off-site Samples can be stored and run together in one assay Maximal respiration can be measured in homogenates and normalized to mitochondrial content
Drawbacks	Samples have to be processed and run on the same day Mitochondrial isolation requires sufficient starting material Control and patient samples cannot always be run simultaneously increasing variability	Samples are not coupled and ATP synthesis capacity cannot be assessed Broken mitochondria that do not maintain mito-chondrial morphology or membrane integrity Metabolic pathways cannot be assessed TCA cycle and transporters are not active

## Data Availability

Data available upon request.

## Abbreviations

Frequently Used Abbreviations: AA: Antimycin A; ADP: Adenosine diphosphate; Asc: Ascorbate; ATP: Adenosine triphosphate; CI: Complex I; CII: Complex II; CIII: Complex III; CIV: Complex IV; CV: Complex V; DH: Dehydrogenase; DNA: Deoxyribonucleic acid; ECAR: Extracellular acidification rate; ETC: Electron transport chain; FCCP: Carbonyl cyanide p-trifluoromethoxyphenylhydrazone; LCL: Lymphoblastoid cell line; LHON: Leber optic atrophy; NADH: Reduced nicotinamide adenine dinucleotide; nDNA: Nuclear DNA; mtDNA: Mitochondrial DNA; MTDR: MitoTracker Deep Red; OCR: Oxygen consumption rate; Oligo: Oligomycin; OXPHOS: Oxidative phosphorylation; PBMC: Peripheral blood mononuclear cell; RIFS: Respirometry in frozen samples; ROS: Reactive oxygen species; Rot: Rotenone; Succ: Succinate; TCA: Tricarboxylic acid cycle; TMPD: N, N, N’,N’-tetramethyl-p-phenylenediamine.
